# Impact of a high dietary fiber cereal meal intervention on the progression of liver fibrosis in T2DM with MASLD

**DOI:** 10.3389/fendo.2025.1623136

**Published:** 2025-09-01

**Authors:** Xi-Shuang Chen, Hui-Zhen Liu, Fang Huang, Jian Meng, Jing-Xian Fang, Yu Han, Hui-Ming Zou, Qing Gu, Xue Hu, Qian-Wen Ma, Yue-Xia Han, Sui-Jun Wang

**Affiliations:** Yangpu District Shidong Hospital of Shanghai, Endocrinology and Metabolism, Shanghai, China

**Keywords:** diabetes, nutritional intervention, liver fibrosis, transient elastography, fibrosis-4 index (FIB-4)

## Abstract

**Aims:**

This study aimed to investigate the ameliorative effect of dietary fiber on hepatic fibrosis in T2DM combined with MASLD and to seek the appropriate dose of DF for this population.

**Material and methods:**

This study was a randomized, controlled, open clinical trial. Patients with T2DM and MASLD were recruited from January 2024 to March 2024 in our hospital using the Interactive Web Response System (IWRS) in a 1:1:1 ratio randomly divided into 3 groups. The control patients received traditional diabetes education. Based on health education, the intervention group took DF supplements (Shanghai Jiuben Technology Co., Ltd.) daily for 12 weeks. Each packet of the supplement is 50 grams and contains approximately 12 grams of dietary fiber (DF). The intervention group was randomly assigned to two different groups. One group received 24 grams of fiber, which was obtained by consuming two packets, each containing 12 grams of fiber. The other group received 12 grams of fiber by consuming one packet.

**Results:**

Both interventions showed a more significant decrease in HbA1c levels than the control group, but only the difference between the control and 24g intervention groups was statistically significant(-0.6 ± 0.5 *vs* -1.6 ± 0.6, P<.001). The 24g intervention group showed a substantial decrease in FPG compared to the control group and the 12g intervention both at week 8 and week 12 (p < 0.05). Both intervention groups experienced significant reductions in FIB-4 levels (p<0.05), with the intervention with 24g group showing the most significant decrease.

**Conclusion:**

Our study demonstrated that dietary fiber improved liver fibrosis and glycemic control, with a more pronounced effect in patients from the 24g Intervention group. However, this group had no statistically significant change in BMI, possibly due to higher caloric intake from additional fiber.

## Introduction

Metabolic Dysfunction-Associated Steatotic Liver Disease (MASLD) is a liver disease associated with metabolic syndrome, characterized by excessive accumulation of fat in liver cells (steatosis), and patients often have metabolic disorders such as obesity, type 2 diabetes, hypertension, or dyslipidemia (1). MASLD is also part of the metabolic syndrome and increases the risk of developing other metabolic diseases (2). The development of type 2 diabetes mellitus (T2DM) is associated with various factors, including genetics, environment, and lifestyle (3). When MASLD coexists with diabetes, a vicious cycle develops between them.

T2DM combined with MASLD not only exacerbates metabolic disorders in patients, but also significantly increases the risk of complications such as cardiovascular disease and chronic kidney disease (4). More seriously, liver fibrosis, a key factor in the progression of MASLD, progresses more rapidly in T2DM patients, leading to serious consequences such as cirrhosis, liver failure, and even liver cancer, posing a serious threat to the health and lives of patients (5).

Insulin resistance plays a central role in hepatic fat deposition and disease progression, and is particularly prominent in the pathogenesis of steatohepatitis (6). The binding of insulin to specific receptors on cell membranes triggers a series of signaling events that promote transmembrane transport of glucose (7). At the same time, this process inhibits pathways involved in glycogen synthesis and lipolysis (8). The mechanism of insulin resistance does not originate solely from the impairment of insulin signaling but is the result of the intertwined and combined effects of multiple metabolic networks (9). The current study shows that free fatty acid (FFA) overaccumulation and hyperinsulinemia reinforce each other, constituting a vicious pathological cycle of MASLD (10). Free fatty acid overload is rooted in caloric surplus, obesity, and decreased insulin sensitivity of adipocytes, leading to enhanced fat mobilization and persistent hyperinsulinemia (11). In addition to lipotoxicity, oxidative stress imbalance, and adipokine secretion disorders, novel factors such as intestinal dysbiosis are involved in MASLD disease progression by activating inflammatory responses (12).

Dyslipidemia serves as a prevalent risk factor for the development of atherosclerosis. Notably, individuals diagnosed with MASLD tend to present a distinctive lipid profile. This particular profile is frequently associated with metabolic syndrome, insulin resistance, and T2DM, characterized by elevated levels of triglyceride-rich lipoproteins, decreased high-density lipoprotein (HDL)-cholesterol, and a higher proportion of small, dense low-density lipoprotein (LDL)-cholesterol particles circulating in the bloodstream (13). Notably, small dense LDL particles have long been recognized for their potent atherogenic properties. Furthermore, epidemiological and genetic investigations have substantiated that elevated plasma triacylglycerol levels causally contribute to an increased risk of cardiovascular disease (CVD), particularly coronary artery disease (14).

For the diagnosis of liver fibrosis progression in patients with T2DM and MASLD, a combination of non-invasive assessment, imaging, serological markers, and pathological examination is required to achieve early identification, dynamic monitoring, and precise intervention.

Currently, the screening and diagnosis of MASLD are often incidental findings, commonly detected during routine physical examinations or when further investigations are conducted due to elevated liver enzyme levels. However, traditional imaging modalities such as conventional ultrasound and computed tomography (CT) have low sensitivity in detecting mild hepatic steatosis, especially when patients also have liver fibrosis or cirrhosis (in such cases, liver fat content may naturally decrease due to disease progression), leading to a higher risk of missed diagnoses and neglecting the high-risk population most in need of early intervention (15).

A liver biopsy is an invasive test that involves certain risks and inconveniences. In recent years, with the advancement of medical technology, some non-invasive diagnostic methods have gradually been widely used, among which the Fibrosis-4 Index (FIB-4) is a highly regarded index for assessing liver fibrosis (16).

In a cross-sectional study by Joo Hyun Oh et al, FIB-4 outperformed other non-invasive models in detecting moderate and advanced fibrosis (17). The FIB-4 is considered a valuable tool in clinical practice for screening patients at high risk of advanced fibrosis due to its simple test format and calculation with the help of a free online calculator (18). However, in at least 30% of cases, the test result will fall within the “uncertain” score range (19).

Transient elastography is a rapid, safe, reproducible LSM (Liver Stiffness Measurement) assessment procedure (20).In a meta-analysis, transient elastography had good diagnostic accuracy for assessing liver fibrosis in patients with MASLD, for whom liver stiffness could be successfully measured (21).

If steatosis is not controlled and treated in time, it may further develop into fatty liver and even lead to serious consequences such as liver fibrosis and cirrhosis (22). MASLD treatment focuses on lifestyle interventions (such as dietary adjustments, regular exercise, and weight loss). Patients with metabolic abnormalities are supplemented with medication (such as insulin sensitizers and vitamin E) or weight loss surgery (23).

Among the many strategies for treating MASLD, weight loss by restricting caloric intake has proven to be one of the most effective approaches (24). This mechanism reduces caloric intake, directly decreases body fat accumulation, and alleviates hepatic steatosis. Recent studies have shown that losing weight through calorie restriction is one of the most effective ways to treat MASLD (25).

Dietary fiber improves body mass index (BMI), HOMA-IR, AST, and ALT in MASLD patients (26). Fiber reduces food intake by stimulating the intestinal secretion of anorexigenic hormones, which can enhance satiety signaling (27). At the same time, fiber slows down carbohydrate absorption, reduces postprandial blood glucose spikes, and indirectly inhibits insulin secretion, thereby reducing fat synthesis triggered by hyperinsulinemia (28). An RCT found that increasing fiber intake (from 19 to 29 g/day of soluble and insoluble fiber) reduced serum zonulin concentrations and significantly improved hepatic enzyme activity in patients with MASLD. This effect may have been achieved by altering intestinal permeability (29).

However, the application of DF to preclinical studies in DM has reported mixed results (30, 31). These differences can be attributed to the source, type, and amount of fiber, the duration of the intervention, and the participants’ age and ethnicity.

Based on this rationale, we conducted a randomized controlled trial to assess the intervention’s effectiveness and sustainability. The primary aim was to investigate the impact of a high-fiber diet on metabolic parameters and liver health outcomes in patients with T2DM and MASLD.

## Methods

### Design of the study

This study was a randomized, controlled, open clinical trial. The study was approved by the Ethics Committee of Shanghai Yangpu Shidong Hospital and completed registration with the China Clinical Trial Registry (ChiCTR1900027663, 11/23/2019).

### Study population

**Figure 1:**
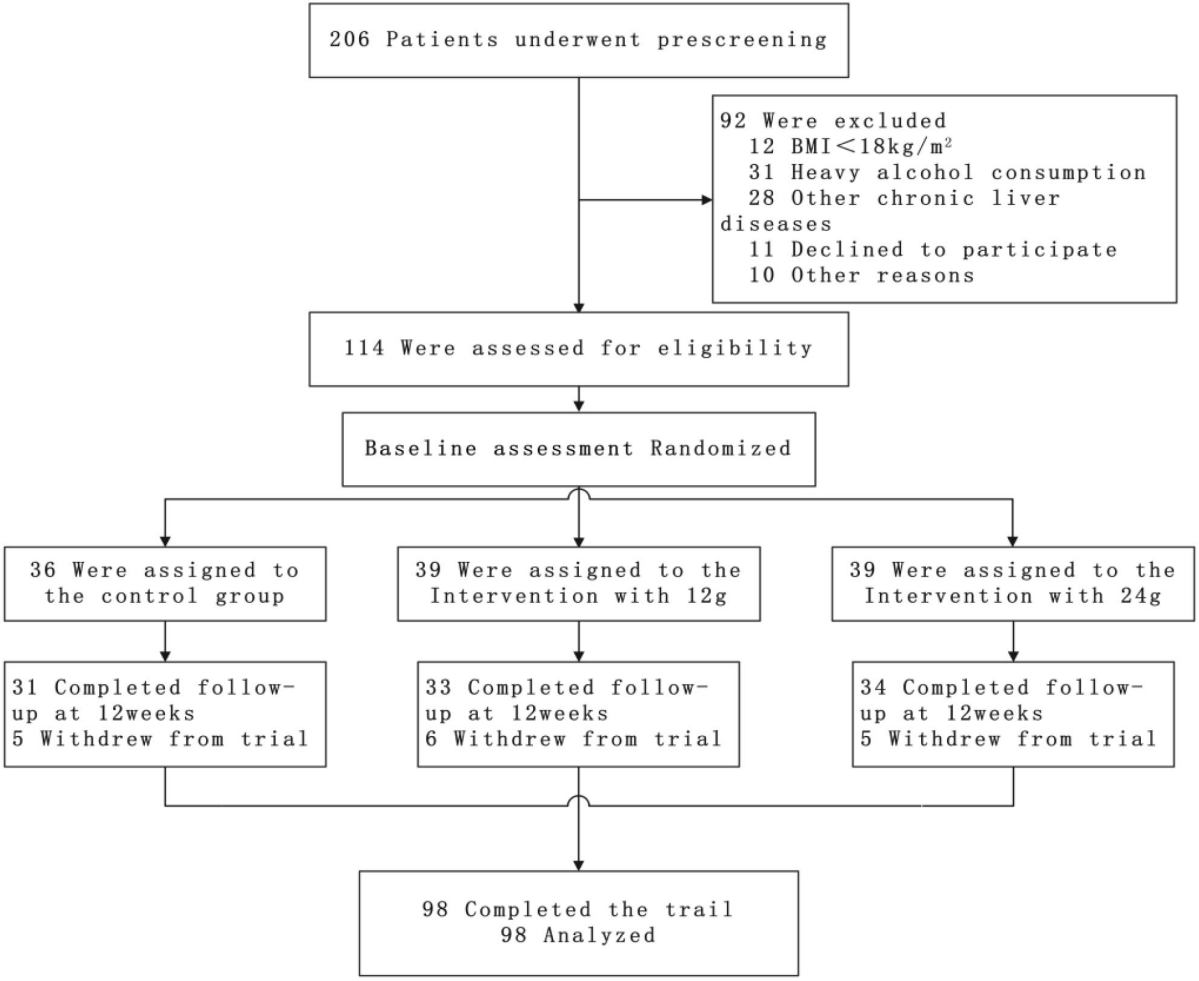
Flow diagram of the study participants.

Patients with type 2 diabetes mellitus (T2DM) and MASLD were recruited from January 2024 to March 2024 in our hospital using the Interactive Web Response System (IWRS) in a 1:1:1 ratio, and were randomly divided into three groups. **Figure 1** provides a flowchart of the recruitment and allocation of study participants.

The inclusion criteria were as follows: (a) aged between 40 and 70 years, (b) presence of T2DM lasting between 1 and 5 years, (c) Body mass index (BMI)≥18kg/m2, (d) T2DM (HbA1c≥6.5% or fasting glucose≥7.0 mmol/L) (32), (e)The diagnosis of MASLD complies with EASL-EASD-EASO Clinical Practice Guidelines (33). MASLD was assessed according to the degree of echo weakening in the liver by color ultrasound examination (34). Liver ultrasound was performed using the same ultrasonographic method as liver disease ultrasonography, aiming to obtain uniform MASLD stratification and to avoid interobserver variations in findings. A liver ultrasound was performed on each study participant by a Toshiba Xario SSA-660A (Toshiba, Japan) device, equipped with a 5 MHz convex probe.

Exclusion Criteria: For patients who drink a lot of alcohol, the amount of alcohol consumed should equate to the amount of alcohol consumed, which is more than 140g/week for men and more than 70g/week for women; T1DM, special type diabetes mellitus, acute complications of diabetes mellitus; pregnant and lactating women; ethanol-induced or drug-induced liver disease, autoimmune disease, viral hepatitis, cholestasis, and metabolic liver disease, hepatomegaly; total parenteral nutrition; autoimmune liver disease; thyroid disease; rheumatoid immune disease.

### Sample size calculation

Changes in glycated hemoglobin in the two intervention groups supplemented with dietary fiber were (-1.1 ± 0.8) and (-1.6 ± 0.6), respectively (35). According to the PASS 15.0 software calculations, a minimum of 30 participants per group is required, considering the 15% dropout rate. With a power of 80% and a two-sided significance level of 0.05, statistical variance tests were conducted on 98 participants.

To address missing data in the study, this study employed multiple imputation based on the random forest algorithm (Multiple Imputation by Chained Equations, MICE), incorporating age, gender, and HbA1c levels as predictor variables, and generated a total of 50 complete data sets to reflect imputation uncertainty.

### Randomization and blinding

After the baseline assessment, patients were randomized to any of the three groups: the control group, the 12g intervention group, or the 24g intervention group, with the help of stand-alone randomization software. To ensure high confidentiality in the randomization process, all randomization operations were performed by a centralized, web-based automated randomization system. To provide a high degree of privacy in the randomization process, all assignments are performed by a centralized, networked, automated randomization system that operates around the clock (24 hours a day). For the specific implementation of random assignment, a static random assignment scheme was used and programmed to operate using the PROC PLAN program in SAS version 9.4 software (provided by SAS Institute Inc). The system relies on a predefined algorithm that generates random sequences with unpredictability, thus ensuring that the distribution of baseline characteristics between the two groups of patients is equalized.

All staff will be divided into three groups based on the needs of the study, and each group will be tasked with guiding the control group, the 12g intervention group, and the 24g intervention group. Strict confidentiality was maintained between the groups, with no disclosure of the specific tasks to each other. In addition, the designers of the study protocol and the staff responsible for the covert grouping and data processing were explicitly excluded from direct participation in the intervention to maintain the objectivity and impartiality of the study.

### Intervention methods

The control patients received traditional diabetes education. The content covered the basic knowledge of diabetes, dietary management, exercise therapy, drug treatment, and blood glucose monitoring. Each session lasted about one hour, and the teaching team consisted of endocrinologists, dietitians, and nurses.

The intervention group took dietary fiber (DF) supplements (Shanghai Jiuben Technology Co., Ltd.) daily for 12 weeks based on health education. Each packet of the supplement is 50 grams and contains approximately 12 grams of dietary fiber. The nutritional information and composition are shown in **Tables 1**, **2**.

**Table 1:** Nutritional information of dietary fiber supplements.

Components	Composition
Carbohydrate (g/100g)	58.6
Protein (g/100g)	11.3
Fat (g/100g)	1.0
Fiber (g/100g)	24.0
Soluble fiber (g/100g)	5.0
insoluble fiber (g/100g)	19.0
Sodium (mg/kg)	800

**Table 2:** Ingredients of the dietary fiber supplement.

Categories	Ingredients								
SDF	Resistant dextrin	Inulin	Oligofructose	Xylo-oligosaccharide					
IDF	Wheat bran	Oat bran	Coicis Semen	Tartary buckwheat	Highland barley	Chia seed	Quinoa	Rye	Millet

SDF, soluble dietary fiber; IDF, insoluble dietary fiber.

The intervention group was randomly assigned to two groups. One group received 24 grams of fiber by consuming two packets, each containing 12 grams of fiber, and the other received 12 grams of fiber by consuming one packet. Take the dietary fiber supplement daily. Add it to 150 ml of warm water, stir well, and drink. Then drink an additional 200 ml of water and eat between breakfast and lunch (10 a.m.).

There were no specific changes in diet (excluding high-fiber packages) or physical activity during the study period. Participants record daily physical activities, including type, duration, and intensity. At the same time, the researchers also telephoned the participants at a set frequency. Throughout the study period, the researchers will also use a diary card to record the participants’ weight data, dietary patterns, bowel movement patterns, medication use, and adverse reactions at regular intervals. It is important to note that all participants were prohibited from using any medication to treat hypoglycemic symptoms or promote weight loss during the intervention.

### Adherence

In the follow-up questionnaire, researchers surveyed participants at 4, 8, and 12 weeks, asking them to report the number of days per week they had supplemented their diet with dietary fiber over the past 12 weeks. Respondents were also asked to keep a daily food diary.

### Anthropometric indicators

Height, weight, and BMI were measured using a body composition scale (OMRON, HNH-318). Waist circumference (WC) and hip circumference (HC) were measured using a circumference tape. During measurement, the subject stood upright with arms hanging naturally at their sides, legs together, abdomen relaxed and held their breath at the end of exhalation. Waist circumference (WC) was measured at the midpoint of the line connecting the lower edge of the ribs and the upper edge of the iliac crest (typically corresponding to the intersection of the umbilical level line and the mid-axillary line). Hip circumference (HC) was measured by wrapping a soft tape measure horizontally around the most prominent point of the hips, snug against the skin but without compression. The waist-to-hip ratio (WHR) and waist-to-height ratio (WHtR) were calculated using the formulas WHR = waist circumference/hip circumference and WHtR = waist circumference/height, respectively.

### Testing indicators

Various vital indicators were carefully observed and recorded before and after the 12-week treatment interval. The primary outcome indicator was HbA1c levels at 12 weeks post-intervention. Secondary indicators include BMI, WC, HC, WHR, WHtR, FBG, HOMA-IR, Ferretin, the Fibrosis-4 score (FIB-4) level, Liver stiffness, and blood lipid profiles (TG, TC, LDL-C, HDL-C) at 8 and 12 weeks of follow-up.

The blood samples were sent to our laboratory within 30 minutes for uniform testing and analysis using a fully automated biochemistry analyzer (BECKMAN COULTER AU5800).

### Assessment of biomarker

All blood samples were collected after 12 hours of fasting and allowed to clot for 1–2 hours at room temperature. 1 to 1.5 mL of venous blood specimens are collected in vacuum tubes containing sodium fluoride for measuring plasma glucose, lipids, and HbA1c. The insulin resistance (HOMA-IR) index was calculated as HOMA-IR = fasting insulin × fasting plasma glucose/22.5.

### Assessment of liver fibrosis

The Fibrosis-4 (FIB-4) index is a noninvasive tool for assessing liver fibrosis (i.e., FIB-4 index = age × AST/[platelet count × (ALT)1/2]). It is easy to use in clinical practice, and its diagnostic ability for advanced liver fibrosis is comparable to magnetic resonance elastography. Transient elastography was used to assess liver stiffness (FibroScan 502 Touch; Echosens). To ensure the accuracy and consistency of MASLD stratification and minimize differences in examination results between observers, a specialist physician with extensive experience in ultrasound diagnosis of liver diseases was specifically assigned to perform the liver ultrasound examination. Specialized probes (such as the M or XL probes from FibroScan) were used, with the appropriate probe selected based on the patient’s body type. The equipment is regularly calibrated to ensure that the measurement error for shear wave propagation velocity is less than 5%.

### Statistical analysis

Data were analyzed using a per-protocol approach. Data were analyzed using SPSS 25.0. Continuous data are expressed as x ¯± s. Multiple group comparisons were analyzed using Analysis of Covariance (ANCOVA). We performed the Shapiro-Wilk test before ANOVA, and the residuals obeyed a normal distribution. Changes in baseline were treated as dependent variables, while group, visit time, and the interaction of group and visit time (group × visit) were treated as independent effects. The Tukey test tested the differences between the groups. Differences were statistically significant at P < 0.05 and significant at P < 0.01.

## Results

### Baseline characteristics of participants

**Figure 1** shows the participant’s flowchart. 114 eligible patients with T2DM combined with MASLD were randomly assigned to the treatment regimen. Of these participants, 98 (86%) completed the 12-week intervention. For the 12-week trial, 5 in the control group, 6 in the Intervention with 12 g, and 5 in the Intervention with 24 g withdrew from the trial, all due to refusal to cooperate. Ultimately, 31 participants in the control group, 33 participants in the Intervention with 12g group, and 34 participants in the Intervention with 24g group completed the trial and were included in the analysis. No significant differences were observed between the three groups (**Table 3**).

**Table 3:** Baseline characteristics of the participants.

Characteristics	CON (n = 31)	Intervention with 12 g (n = 33)	Intervention with 24 g (n = 34)	P
Age, years	61.0 (48.9-68.3)	58.4 (44.7-69.0)	62.7 (47.1-66.8)	0.66
BMI, kg/m2	26.7 (24.4-28.3)	25.1 (23.9-29.0)	26.8 (25.1-28.8)	0.37
WC (cm)	92.1 (81.7-100.6)	93.8 (82.2-101.1)	91.5 (81.9-101.8)	0.53
HC (cm)	99.9 (92.5-107.4)	98.4 (90.2-108.3)	100.8 (91.1-107.9)	0.66
WHR	0.90 (0.81-0.96)	0.90 (0.82-0.95)	0.90 (0.81-0.97)	0.15
WHtR	0.55 (0.51-0.60)	0.56 (0.51-0.61)	0.54 (0.50-0.60)	0.42
Platelet count, ×10^3^/µL	20.6 (18.3-24.9)	22.2 (19.5-25.5)	21.2 (18.5-24.2)	0.74
AST, U/L	44.1 (36.8-59.9)	48.4 (39.5-56.6)	45.8 (35.5-58.1)	0.23
ALT, U/L	57.0 (49.6-68.3)	55.2(44.7-64.7)	59.4 (48.7-66.4)	0.61
GGT, U/L	65.1 (35.0-92.7)	68.9 (39.4-94.8)	68.5 (40.2-99.3)	0.62
TC, mmol/l	6.6 (5.8-6.9)	6.5 (5.5-7.1)	6.3 (5.4-6.9)	0.48
TG, mmol/l	2.6 (2.2-3.0)	2.5 (2.1-3.1)	2.4 (2.0-2.9)	0.33
HDL, mmol/l	1.4 (1.0-1.9)	1.5 (1.2-1.9)	1.3 (1.0-1.8)	0.45
LDL, mmol/l	3.8 (3.0-4.1)	3.7 (3.3-4.2)	3.6 (3.1-4.1)	0.33
FPG, mmol/l	8.1 (5.8-9.7)	7.7 (6.0-9.1)	8.0 (6.2-8.8)	0.41
HbA1c, %	7.5 (6.8-8.0)	7.2 (6.6-8.2)	7.3 (6.5-8.3)	0.79
HOMA-IR, median (IQR)	3.6 (2.5-6.2)	3.4 (2.6-6.0)	3.8 (2.9-6.2)	0.66
Ferritin, ng/mL	243 (167-304)	225 (179-300)	251 (172-298)	0.58
Liver stiffness, kPa	6.6 (5.3-8.1)	6.5 (5.6-8.4)	6.7 (5.7-8.8)	0.72
FIB-4	1.6 (0.9-2.1)	1.8 (1.2-2.2)	1.6 (0.8-2.0)	0.61
Intake				
Energy, kcal/d	2321.7 (2037.0-2511.8)	2278.4 (2015.8-2627.9)	2307.5 (2127.6-2612.4)	0.38
Fat, %	36.5 (30.7-40.8)	34.4 (31.9-39.9)	33.7 (30.2-41.2)	0.53
Protein, %	15.9 (12.8-17.9)	14.6 (11.9-18.5)	15.0 (11.3-19.3)	0.31
Carbohydrate, %	46.3 (38.9-50.1)	44.5 (39.8-51.1)	44.8 (37.9-49.9)	0.74
Alcohol, median (IQR),g/wk	0.0 (0.0-6.2)	0.0 (0.0-5.7)	0.0 (0.0-5.5)	0.59
Physical activity, median (IQR), MET/wk	12.4 (6.6-20.2)	10.8 (6.9-19.5)	13.3 (7.7-18.6)	0.39

Values are expressed as median (upper quartile, lower quartile).

BMI, body mass index; AST, aspartate aminotransferase; ALT, alanine transaminase; GGT, gamma-glutamyl transferase; TC, total cholesterol; TG, triglycerides; HDL, high density lipoprotein; LDL, low density lipoprotein; FPG, fasting plasma glucose; HbA1c, glycated hemoglobin; FIB-4, Fibrosis-4 score.

Differences between groups were not significant (all P > 0.05).

### Glucose-related indicators

Both interventions showed a more significant decrease in HbA1c levels compared to the control group. Still, only the difference between the control group and the 24g intervention group was statistically significant(-0.6 ± 0.5 *vs* -1.6 ± 0.6, P<.001) (**Table 4**).

**Table 4:** Summary of posthoc multiple comparison analyses change at 12 weeks.

Outcome variables	CON (n = 31)	Intervention with 12 g (n = 33)	Intervention with 24 g (n = 34)	p Value* group–time interaction	CON *vs* intervention with 12 g	CON *vs* intervention with 24 g
WC (cm)	-0.8 ± 0.4	-0.6 ± 0.6	-2.7 ± 0.4	0.034	0.562	0.032
HC (cm)	-1.1 ± 0.5	-0.9 ± 0.4	-0.9 ± 0.5	0.336	0.324	0.644
WHR	0.02 ± 0.02	-0.01 ± 0.02	-0.02 ± 0.03	0.352	0.116	0.747
WHtR	-0.02 ± 0.03	-0.02 ± 0.03	-0.02 ± 0.04	0.712	0.558	0.412
AST, U/L	-3.2 ± 1.9	-6.7 ± 1.3	-10.4 ± 2.1	0.022	0.571	<.001
ALT, U/L	-2.5 ± 2.2	-3.8 ± 1.7	-9.1 ± 2.0	0.013	0.583	<.001
GGT, U/L	-5.3 ± 2.3	-5.8 ± 1.3	-11.4 ± 2.9	0.011	0.332	<.001
TC, mmol/l	-0.9 ± 1.1	-1.8 ± 0.8	-1.9 ± 1.2	0.745	0.085	0.012
TG, mmol/l	-0.6 ± 0.6	-1.6 ± 0.9	-1.8 ± 0.9	0.011	0.026	<.001
HDL, mmol/l	-0.3 ± 0.1	-0.5 ± 0.2	-0.6 ± 0.3	0.332	0.648	0.451
LDL, mmol/l	-0.2 ± 0.3	-0.3 ± 0.3	-0.3 ± 0.2	0.578	0.412	0.748
HbA1c, %	-0.6 ± 0.5	-1.1 ± 0.8	-1.6 ± 0.6	0.337	0.386	<.001
HOMA-IR, median (IQR)	-0.9 ± 0.4	-1.2 ± 0.5	-2.5 ± 0.3	0.014	0.331	<.001

The values were presented mean ± SD.

AST, aspartate aminotransferase; ALT, alanine transaminase; GGT, gamma-glutamyl transferase; TC, total cholesterol; TG, triglycerides; HDL, high density lipoprotein; LDL, low density lipoprotein; FPG, fasting plasma glucose; HbA1c, glycated hemoglobin.

*p Values for the comparison of the value at the follow-up time with the baseline value within the group, as calculated with the use of mixed-model repeated measures analysis of variance.

**Figure 2:**
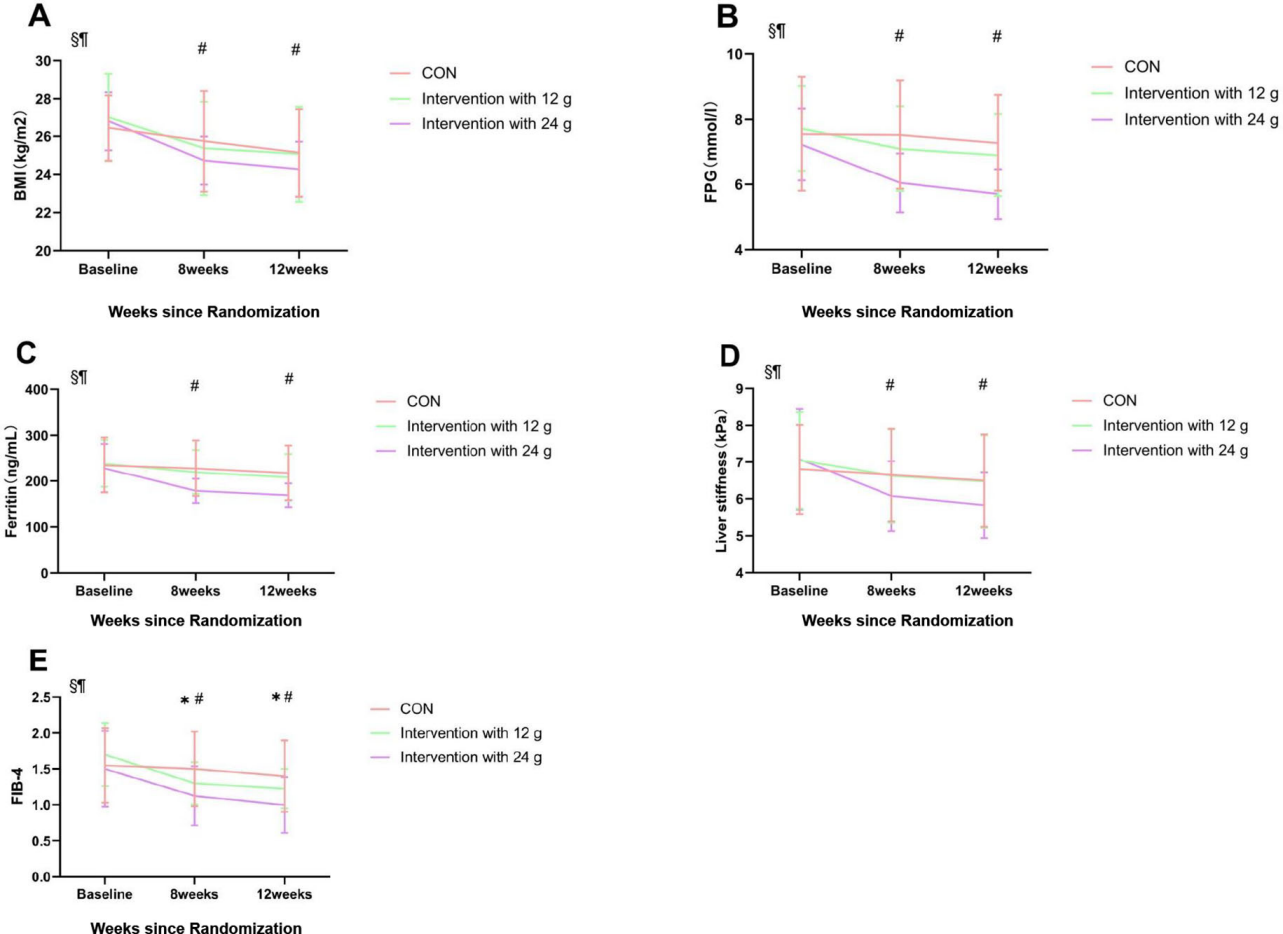
Contrast results from repeated ANOVA. Comparison between groups at 8 and 12 weeks postintervention. Mean changes in BMI **(A)**, fasting plasma glucose concentration (FPG) **(B)**, Ferritin **(C)**, Liver stiffness **(D)**, and FIB-4 **(E)** in the CON, Intervention with 12g and Intervention with 24g groups from baseline are shown. The points correspond to the estimated means from the repeated measures ANOVA model. *Indicates statistically significant difference between CON and Intervention with 12g, p < 0.05. ^#^Indicates statistically significant difference between CON and Intervention with 24g, p < 0.05. ^§^p < 0.05 for the effect of time; ^¶^p < 0.05 for the interaction between groups and time. Data are presented as the mean and SD. CON, control exercise.

The 24g intervention group showed a significant decrease in FPG compared to the control group and the 12g intervention group at both week 8 and week 12 (p < 0.05) (**Figure 2B**).

### Anthropometric indicators

The Group-Time interaction effect of BMI was significantly different. After 8 and 12 weeks, a significant difference in BMI was observed between the control group and the 24g intervention group. No significant difference in BMI was found between the 12g intervention group and the control group (**Figure 2A**).

After 12 weeks, WC (P = 0.034) was lower than baseline in the 24g intervention group (**Table 4**).

### Changes in the degree of insulin resistance

The Group-Time interaction effect (P = 0.014) for HOMA-IR was significant. HOMA-IR levels in the intervention with the 24g group (P<.001) were significantly lower than in the control group after 12 weeks. The intervention with the 12g group showed a slight but not statistically significant improvement in HOMA-IR (**Table 4**).

### Liver fibrosis-related indicators

The intervention with the 24g group experienced significant reductions in ferritin levels both at week 8 and week 12 (p<0.05). There was no significant difference in ferritin reduction between the control and 12 g intervention groups. (**Figure 2C**).

Significant liver stiffness reductions were observed in the intervention group with 24g (p<0.05), while there were no substantial changes in the other groups. (**Figure 2D**).

Both intervention groups experienced significant reductions in FIB-4 levels (p<0.05), with the intervention group with 24g showing the most significant decrease (**Figure 2E**).

In the intervention with the 24g group, we observed a significant reduction in AST, ALT, and GGT (P<.001) (**Table 4**).

### Lipid-related indicators

The Group-Time interaction effect (P = 0.011) for TG was significant. After 12 weeks, there was a significant decrease in TG in the 12g intervention group (P = 0.026) and the 24g intervention group (P<.001) compared to the control group. Both intervention groups showed decreases in TC levels compared to the control group, but the difference was significant only for the 24g intervention group when compared separately (P = 0.012) (**Table 4**).

### Adherence

Among the participants, the proportions who responded to the follow-up questionnaire and achieved the recommended intake of dietary fiber five or more times per week at each follow-up were 92.7%, 90.4%, and 89.1%, respectively.

### Adverse reaction

During the intervention implementation phase, three participants in the 24g intervention group reported abdominal bloating, which mainly occurred within 1–3 hours after taking dietary fiber. The severity was described as “mild discomfort that was tolerable” and did not affect daily activities. No other severe adverse reactions were recorded in the control group and the 12g intervention group.

## Discussion

This randomized clinical trial provides new findings on the effect of a high-fiber diet on Type 2 Diabetes Mellitus (T2DM) combined with Metabolic Dysfunction-Associated Steatotic Liver Disease (MASLD). Our intervention involved providing patients with additional doses of fiber in varying amounts, specifically 12 and 24 grams. The results of the biochemical study revealed improved glycemic control and obesity in patients who consumed 24 grams of fiber. Additionally, a high-fiber diet exacerbates the degree of steatosis and fibrosis in MASLD.

### Effect of dietary fiber on glucose-related indicators

Dietary fiber can be classified into two main categories based on its solubility in water: soluble dietary fiber and insoluble dietary fiber (36). Insoluble dietary fiber is predominant in whole grains, such as wheat bran, and constitutes the coarse fiber structure of the outer layer of grains. When mixed with gastric juice, insoluble fiber absorbs water and expands to 3–5 times its original volume, forming a high-viscosity gel-like substance that significantly slows gastric emptying, thereby reducing postprandial glucose (PPG) and insulin levels (37).

Soluble fiber, primarily consisting of β-glucan, pectin, and resistant dextrin, forms a high-viscosity solution in the stomach and small intestine. By coating starch particles and inhibiting the activity of α-glucosidase, it directly slows down the rate of glucose production (38).

The results of our study suggest that increasing dietary fiber intake is beneficial for glycemic control in T2DM. The 24g intervention group achieved superior results in glycemic management compared to the 12g intervention group. Further research suggests that increasing daily dietary fiber intake from 15 grams to 35 grams may be beneficial for HbA1c levels in people with diabetes (39). In an RCT study of MASLD, there was a trend toward less hepatic steatosis in the group consuming 24 g of fiber per day compared to the group consuming 12 g of fiber per day (p = 0.07) (40).

The mechanism may be that a high-fiber diet helps promote intestinal peristalsis, maintains intestinal ecological balance, lowers postprandial blood glucose levels, improves insulin sensitivity, and reduces blood glucose fluctuations (38). Zhao and other researchers have revealed that the gut microbiota can provide additional health benefits to patients with T2DM through carbohydrate fermentation and the production of short-chain fatty acids (SCFAs) (41). In another systematic review, foods rich in soluble fiber, such as β-glucan, improved blood glucose levels in diabetic patients (42). Moreover, a high intake of dietary fiber promotes satiety. It reduces the intake of high-energy foods, thereby reducing the risk of overweight/obesity, an estimated risk factor for T2DM (30).

### Effect of dietary fiber on body mass index

A meta-analysis reported a significant dose-response relationship between body mass index and MASLD risk (43). In our study, we found a dose-dependent effect of dietary fiber on improving BMI. Although the mechanisms underlying the association between obesity and MASLD are not fully understood, the adipose tissue (AT) distensibility hypothesis could link obesity to the development of MASLD (44). Obesity, a growing health problem, is emerging as a significant challenge worldwide. Unhealthy eating habits are an essential factor in the development of obesity. With the accelerated pace of life and changes in dietary patterns, people are increasingly inclined to choose foods high in calories, fat, and sugar. Dietary fiber can have a positive effect on the prevention and treatment of obesity through a variety of mechanisms, including increasing satiety, lowering the glycemic index, promoting intestinal health, regulating fat metabolism, and lowering cholesterol (45). Studies have shown that increased intake of vegetables, fruits, legumes, and diets high in soluble fiber can reduce body weight and body fat percentage (46). In our study, both groups in the dietary fiber intervention had a significant BMI reduction compared to the control group. Still, the difference in BMI between the 12g intervention group and the 24g intervention group was insignificant.

This study did not systematically record participants’ energy and nutrient intake during the intervention period, making it impossible to directly assess the contribution of dietary structure changes to the intervention’s effectiveness. Participants may have spontaneously reduced their intake of high-calorie foods due to increased health awareness resulting from the intervention, and this behavioral change was not captured in this study, leading to an overestimation of the independent effect of the high dietary fiber cereal meal intervention.

### Effect of dietary fiber on lipid-related indicators

One study showed that 23.76 grams of combined dietary fiber content per day resulted in a significant decrease in total cholesterol (TC) and low-density lipoprotein cholesterol (LDL-C) compared to a conventional diet (47). This study is similar to our findings. This result may be due to the key role played by the viscous properties of DF, which delay gastric emptying, meaning that food stays in the stomach longer, thus slowing down digestion and absorption. In addition, DF inhibits the transit of triglycerides and cholesterol through the intestines. This inhibition helps reduce the amount of these lipids absorbed into the bloodstream by the small intestine, lowering blood lipid levels (48).

### Effect of dietary fiber on MASLD

A cross-sectional study of 265 healthy adults and animal experiments found that individuals in the highest quartile of vegetable intake had lower rates of ALT abnormalities. Additionally, supplementation with insoluble/soluble dietary fiber significantly reduced serum ALT/AST levels, and increased serum albumin and total protein levels (49). Another study further confirmed that dietary fiber intake was positively correlated with improvements in MASLD biochemical indicators (50). These are similar to our research findings.

The mechanism by which dietary fiber affects MASLD may be that its well-known role in modulating the gut microbiota is an integral part of the gut barrier (51). When the gut barrier is compromised, the liver is the first parenchymal organ to be exposed to bacteria and bacterial products, which can lead to inflammation and liver damage (52). Second, Short-Chain Fatty Acids (SCFAs) produced by the gut microbiota fermenting dietary fiber prevent MASLD (53). In addition, by modulating the gut microbiota and metabolite production, dietary fiber may inhibit the expression of inflammatory factors in the liver and reduce the inflammatory response (54). This improvement may be associated with fiber, oligofructose, inulin, and oligosaccharides. Oligofructose, inulin, and oligo-xylulose selectively promote the growth of bifidobacteria (55). The meta-analysis findings revealed statistically significant correlations between MASLD and C-reactive protein (CRP), interleukin-1β (IL-1β), interleukin-6 (IL-6), tumor necrosis factor-α (TNF-α), and intercellular adhesion molecule-1 (ICAM-1), with corresponding odds ratios (ORs) of 1.41, 1.08, 1.50, 1.15, and 2.17, respectively (56). In this study, participants in the 24g intervention group demonstrated a statistically significant decrease in ferritin levels at both week 8 and week 12 of the intervention period (p<0.05).

Compared with traditional dietary approaches, bagged fiber foods have significant advantages in diabetes-combined MASLD populations and effectively boost daily fiber intake. First, the convenience of fiber-in-bag foods is one of their major highlights. In addition, the fiber in fiber-in-a-bag foods is derived from various high-quality grains, making them more nutritious and diverse.

In real life, consuming 24 grams of dietary fiber daily is feasible; however, individual differences and nutritional adjustments should be taken into account. Maintaining this intake level over the long term can provide health benefits, but excessive intake should be avoided to prevent potential risks. Excessive fiber intake (>50 grams/day) may reduce the absorption of minerals such as iron, zinc, and calcium, but the risk is low at 24 grams. Sudden increases in fiber intake may cause bloating or diarrhea, and adequate hydration can help alleviate these symptoms (57).

In summary, long-term dietary fiber intake should be adjusted according to individual health conditions, age, and special needs in terms of intake methods and food choices.

## Limitations

First, since the study period was only 12 weeks, which is relatively short, it was somewhat difficult to conclude long-term outcomes, such as the progression of fibrosis and cardiovascular outcomes. Second, DF supplements contain both soluble and insoluble fiber components, which could have varying impacts on glycemic control and lipid metabolism. However, there is still a lack of clear understanding of the respective contributions of these two components and whether synergistic effects exist. Future research needs to clarify the optimal ratios of various types of fibers, for example, by comparing the effects of soluble versus insoluble fibers on blood glucose, lipids, and hepatic steatosis in randomized controlled trials. Third, patients’ dietary reports may not objectively and truthfully reflect dietary information, and patients may report inaccurate nutritional information due to fuzzy memory or forgetfulness, resulting in inconsistent data and affecting the accuracy and reliability of the study. Fourth, in addition to conventional methods such as telephone follow-ups and food diaries, there is currently a lack of clear and widely accepted strategies for verifying dietary fiber intake compliance. This situation may adversely affect the accuracy and validity of research results. Fifth, insufficient sample size is a significant limitation of this study. The small sample size made it impossible to conduct subgroup analysis according to the established statistical requirements, thereby preventing in-depth exploration of the relationship between subgroup characteristics, such as gender differences and fibrosis stages, and related variables.

This study did not systematically record participants’ energy and nutrient intake during the intervention period, making it impossible to directly assess the contribution of dietary structure changes to the intervention’s effectiveness. Previous studies have shown that approximately 30% of weight loss in behavioral interventions can be attributed to spontaneous reductions in energy intake (58). This factor may partially confound the positive results of this study. Participants may have spontaneously reduced their intake of high-calorie foods due to increased health awareness resulting from the intervention, and this behavioral change was not captured in this study, leading to an overestimation of the independent effect of the high dietary fiber cereal meal intervention.

We could not elucidate a direct causal relationship between a high-fiber diet and liver fibrosis in MASLD. Moreover, although our statistical analyses adjusted for several potential confounding variables, the possibility of residual or unmeasured confounders could not be excluded entirely.

Analyzing only subjects who completed the intervention may have excluded patients who withdrew due to adverse effects or inefficacy, leading to biased results in favor of the intervention group. In the current study, those who dropped out did so due to refusal to cooperate, not because of exacerbation. Patients who completed the study had relatively better glycemic and weight control or were more cooperative with treatment, so this study may have overestimated the effect of a high-fiber diet.

## Conclusions

Our study demonstrated that dietary fiber improved liver fibrosis and glycemic control, with this effect being more pronounced in patients in the 24g Intervention group. However, this group showed no statistically significant change in BMI.

From a clinical application perspective, these results carry substantial weight. They suggest that dietary fiber intervention, especially at a daily dose of 24 g, holds promise as a therapeutic strategy for managing liver fibrosis and glycemic dysregulation. Clinicians can consider incorporating appropriate dietary fiber supplements into the treatment plans of patients with liver-related conditions and diabetes, aiming to achieve better disease control. Yet, the lack of BMI reduction despite fiber intake underscores the need for clinicians to closely monitor patients’ overall caloric intake when recommending fiber-rich diets or supplements. This ensures a comprehensive approach to patient care, balancing the benefits of fiber on liver and glycemic health while preventing potential weight-related issues due to excessive calorie consumption. For patients with diabetes and MASLD, in addition to conventional drug treatment and lifestyle guidance, increasing dietary fiber intake can be considered as an adjunctive treatment.

Given the limitations of a 12-week study period in drawing long-term conclusions about the progression of liver fibrosis, future studies should extend the study duration. Long-term follow-up studies spanning several years could be designed. To address the issue of inaccurate dietary reporting, future studies could adopt a multi-method approach. In addition to traditional methods such as telephone follow-ups and dietary diaries, new technologies should be integrated. For example, mobile applications can be used to enable patients to record their dietary intake in real-time, and scanning barcodes on packaged foods can ensure the accuracy of nutritional information. The sample size and recruitment areas should be expanded to reduce geographical influences and limitations. Future studies could further explore the specific benefits of different types of dietary fiber for various populations and investigate their long-term effects.

## Data Availability

The original contributions presented in the study are included in the article/supplementary material. Further inquiries can be directed to the corresponding authors.
